# Protocol of a scoping review of systematic reviews and meta-analyses about COVID-19 vaccines and associated adverse events from vaccination

**DOI:** 10.1371/journal.pone.0285442

**Published:** 2023-05-10

**Authors:** Shelly Melissa Pranić, Lenny T. Vasanthan, Jacqueline Y. Thompson, Vinayak Mishra, Pratyush Kumar, Roshan Arjun Ananda, Narges Malih, Ka-King Chan

**Affiliations:** 1 Department of Public Health, University of Split School of Medicine, Split, Croatia; 2 Cochrane Croatia, Split, Croatia; 3 Department of Physical Medicine and Rehabilitation, Christian Medical College, Vellore, India; 4 Institute of Applied Health Research, University of Birmingham, University of Birmingham, Birmingham, United Kingdom; 5 Department of Children’s Health, University of Liverpool, Liverpool, United Kingdom; 6 Baba Saheb Ambedkar Medical College, New Delhi, India; 7 Queensland Health, Queensland, Australia; 8 Global Health Research Group, University of the Balearic Islands (UIB), Palma, Spain; 9 Social Determinants of Health Research Center, Shahid Beheshti University of Medical Sciences, Tehran, Iran; 10 Pamela Youde Nethersole Eastern Hospital, Hong Kong, China; Lagos State University, NIGERIA

## Abstract

The continuous dissemination of coronavirus disease of 2019 (COVID-19) literature can inform decision-makers and the public. Since the widespread use of COVID-19 vaccines, more systematic reviews have summarized the effectiveness and reported adverse events associated with vaccination. Previous systematic and scoping reviews on COVID-19 summarized various aspects surrounding COVID-19, however, a scoping review is needed to summarize the characteristics of COVID-19 vaccines and associated adverse events reported in systematic reviews and meta-analyses to provide comprehensive evidence for informed medical decision-making. We will conduct a scoping review concerning COVID-19 vaccines and adverse events from vaccines. We will search from December 2019 to present in Epistemonikos, Campbell Library, CINAHL (Ovid), MEDLINE (Ovid), Scopus, CENTRAL (Ovid), Web of Science, WHO COVID-19 database, Joanna Briggs Institute of Excellence, and COVID-19 Evidence Reviews resource. We will include systematic reviews, meta-analyses, or both of randomized controlled trials and observational studies and exclude individual randomized controlled trials and observational studies. Abstracts and full-texts will be screened prior to selection. Investigators will independently use a calibrated quantitative and qualitative data extraction sheet and rate the quality of articles with AMSTAR, resolving disagreements to aim for good agreement (≥80%). An updated scoping review of the characteristics and safety of COVID-19 vaccines would highlight the accuracy of the evidence to inform decision-making concerning COVID-19 vaccination.

## Introduction

Coronavirus disease 2019 (COVID-19) continues to fuel scientific publication [1]. The amount of published information about COVID-19 is continuously increasing, with over a tenfold increase in the volume of publications in a short time span, from 56,534 in December 2020 to 856.395in April 2023 [2, 3]. Considering the abundance of information about COVID-19, it may be difficult for clinicians and decision-makers to find which studies about COVID-19 may have sufficient methodological rigor to provide definitive answers to clinical questions about different factors surrounding the disease. There have been concerns about the quality of reporting in the published studies on COVID-19, as highlighted by researchers [1, 4, 5]. Recently, multiple systematic reviews have provided an overview of the safety and effectiveness of COVID-19 vaccines in diverse settings [6–20]. The quality of this new evidence from systematic reviews and meta-analyses on COVID-19 vaccines and vaccine-associated adverse events have yet to be examined to inform medical decision-making. Thus, a summary of the characteristics of COVID-19 vaccines and their reported adverse events along with an assessment of the quality of the evidence would add to the strength of the existing evidence, which will be of great importance to public health.

The objective of our scoping review is to summarize the characteristics of information/evidence dissemination that will improve medical decision-making. This scoping review will supplement a future study to be conducted by our group which is to identify systematic reviews that could facilitate the evaluation of the accuracy of COVID-19 vaccine and vaccine-associated adverse events information intended for the public.

### Objectives

1. Gather high-quality evidence on which experimental and observational studies have reported about COVID-19 vaccines and adverse events associated with COVID-19 vaccines.
2. Identify high-quality evidence about COVID-19 vaccines and adverse events related to vaccines.
3. Identify areas in the research enterprise where there is some evidence about COVID-19 vaccines and adverse events associated with COVID-19 vaccination.
4. Provide the most definitive evidence to determine what information is currently accurate about COVID-19 vaccines and vaccine-related adverse events.

## Materials and methods

The scoping review protocol is registered at the Open Science Framework (https://osf.io/gkv74/?view_only=f93fe736a60747e09d3c7d442a49e1bc). We reported this scoping review in accordance to the Preferred Reporting Items for Systematic Reviews and Meta-Analyses Protocols (PRISMA-P) statement and completed the corresponding checklist [21] (S1 File). We will follow the PRISMA extension for scoping reviews (PRISMA-ScR) guidelines to report this scoping review [22]. Additionally, we will follow guidance on establishing the framework for conducting scoping reviews from Covidence and the Joanna Briggs Institute (JBI) [23]. We chose the COVID-19 search terms for this scoping review based on a previous study that assessed the main concepts in the media concerning COVID-19 [24]. Consequently, the investigators, consisting of evidence-based healthcare researchers in health services research, medicine, and public health, chose themes that pertain to our current evaluation and added relevant COVID-19 disease aspects to the research question.

Our research question is, “what is the available evidence on COVID-19 vaccines and associated adverse events from COVID-19 vaccines reported in systematic reviews and meta-analyses?” As the development of vaccines has come to the forefront in preventing COVID-19, we have chosen to focus our scoping review on vaccines and adverse events arising from their use. This research question would guide our scoping review to summarize the evidence on the growing body of systematic reviews on COVID-19 vaccines and related adverse events. Further, a comprehensive summary of systematic reviews of the evidence about COVID-19 vaccines and their reported adverse events would provide descriptions of their effectiveness and safety to inform the public and policy makers.

Accordingly, we plan to apply evidence-based approaches to conduct our scoping review, of which the first step was completed by identifying the research question found above. Additionally, we will state the eligibility criteria, conduct searches for evidence using a defined search strategy, select relevant systematic reviews, extract evidence, chart the evidence, and summarize and present our findings [23].

### Literature search and strategy

Our methods were described in detail in our protocol posted on Open Science Framework (https://osf.io/gkv74/?view_only=f93fe736a60747e09d3c7d442a49e1bc). We will conduct a comprehensive search of literature sources according to guidelines from the JBI reviewers’ 2015 manual for scoping reviews and using COVID-19 search filters from the Canadian Agency for Drugs and Technologies in Health (CADTH) [23, 25]. A medical librarian will provide advice regarding writing the search strategy. Further, most of our search strategy was peer-reviewed by a medical information specialist in November 2021 using the Peer Review of Electronic Search Strategies (PRESS) tool to validate the search strategy [26]. Subsequent versions of the protocol will reflect the revisions suggested by the PRESS evaluation. Following JBI guidelines, obtaining advice from a librarian, and validating our search with the PRESS tool, we intend to conduct a replicable and methodologically robust scoping review and search strategy. Our draft search strategy for Epistemonikos, Scopus, MEDLINE (Ovid), CINAHL (Ovid), CENTRAL (Ovid), Web of Science (Advanced search), WHO COVID-19 database, and the Joanna Briggs Institute of Excellence is available in the S2 File.

### Sources of information

Sources will include published systematic reviews with or without meta-analyses describing the effectiveness and safety of vaccines to prevent COVID-19 disease. We will search electronic bibliographic databases (Epistemonikos, Campbell Library, Cumulative Index to Nursing and Allied Health Literature (CINAHL) (Ovid), Pubmed/MEDLINE (Ovid), Scopus, Cochrane Central Register of Controlled Trials (CENTRAL) (Ovid), Web of Science Core Collection, the WHO COVID-19 database, Joanna Briggs Institute of Excellence, and the COVID-19 Evidence Reviews resource) from January 1, 2019 to the present date. We will use the Ovid platform to search in the MEDLINE, CINAHL, and CENTRAL databases. Relevant preprint servers (e.g., MedArxiv and bioRxiv) and the gray literature will also be searched.

The keywords for the searches in electronic databases, preprint servers, and the gray literature will be derived from our research question to find relevant articles eligible for inclusion in our studies. We will revise and update our scoping review research question, eligibility criteria, or search strategy if needed due to new information discovered during the literature searches and advice from peer review and the librarian.

Regarding the current stage of this scoping review, we have only performed preliminary searches in the databases and have not proceeded to the study selection phase.

### Study eligibility criteria

#### Inclusion and exclusion criteria

We will include systematic reviews with or without meta-analyses of randomized controlled trials (RCTs), and observational, non-interventional studies (cohort, cross-sectional, case-control, case-series, case-studies). The rationale for including systematic reviews of RCTs is that RCTs have the best study design to determine the safety and efficacy of vaccines. Systematic reviews of observational studies would best describe the follow-up of participants who have received at least one dose of a COVID-19 vaccine using non-interventional methods, contributing to this scoping review as some research questions can only be addressed without performing any intervention. The RCTs should compare COVID-19 vaccines with a placebo or no comparisons. Additionally, we will include systematic reviews of trials that summarize COVID-19 vaccines used as the exposure, adverse events from vaccines, and patient-centered outcomes including hospitalization or intensive-care unit (ICU) admission. We will contact study authors if there is a lack of information to determine eligibility.

We will exclude (1) duplicate publications, (2) individual randomized controlled trials or observational studies, (3) reviews without sufficient descriptions or results (protocols, conference proceedings, abstracts, letters, editorials, ongoing or unpublished studies, or commentaries), (4) systematic reviews that do not report the quality/risk of bias of the included primary studies, and (5) systematic reviews not available in English.

If several systematic reviews summarize the same element to assess accuracy, we will consider the single publication with the highest A Measurement Tool to Assess Systematic Reviews-2 (AMSTAR-2) quality score to appraise methodological shortcomings in systematic reviews with or without meta-analyses. The quality assessed by the AMSTAR-2 will be categorized as critically low, low, moderate, or high quality independently by the investigators. We will allot numerical scores for responses to the AMSTAR-2 questions of 1, 0.5, and 0 points for “yes,” “partial yes,” and “no.” The minimum total AMSTAR-2 score would be 0. Systematic reviews with meta-analyses will have a maximum of 7 points. Systematic reviews without meta-analyses will have a maximum of 5 points [27].

We will discuss unclear eligibility amongst the reviewers until consensus is reached for those cases with unclear eligibility.

### Identification and selection of reviews

This scoping review involves a team of healthcare professionals with expertise across health services research, medicine, and public health.

A single reviewer (SP) will screen all citations and remove duplicates using EndNote (version X9, Clarivate, Philadelphia, USA). Before full data extraction, each team member will extract data from identical systematic reviews to calibrate and amend the inclusion criteria and extraction if needed. We will repeat calibration phases to ensure that extractions follow the eligibility criteria and further calibrate the extraction sheet. After each calibration, we will assess agreement in the study design, AMSTAR-2 quality, and extracted data. We will aim for reliability coefficients (intra-class correlation coefficient [ICC]) > 0.80 before proceeding to the main extraction. Any disagreements will be resolved through consensus discussion.

Each team member will then independently screen the titles, abstracts, and full text of the articles from a batch of the systematic reviews unique to each reviewer. We will review the reference lists for additional studies not found by our initial search. We will describe included and excluded studies with a flow chart during the various stages of the scoping review.

### Data collection and extraction

After the calibration period described above, the remaining systematic reviews will be divided into similarly sized batches using EndNote: (1) the publication date of the records will be sorted in chronological order (oldest to newest), (2) then we will use the search function in EndNote to divide the records into batch sizes that will be similar in size, (3) then the batches will be exported to Rayyan [28] as separate files using the RIS export option. Each investigator will then independently determine the eligibility of the systematic reviews for inclusion.

Aligned with the objectives concerning gathering evidence, for this scoping review, all reviewers will extract relevant data on COVID-19 vaccines and vaccine-related adverse events that will allow an assessment of the data to provide a subsequent comprehensive summary from the systematic reviews. We will extract data from systematic reviews including the author, dates of publication (year), publication title, journal-level characteristics including journal title and impact factor, study design characteristics, details about the population including sociodemographic characteristics, days spent hospitalized or in the ICU), sample size, types of vaccines for the prevention of COVID-19, the level of vaccine effectiveness, frequency of adverse events reported after vaccination, comparators, and other reported outcomes. Additionally, we will extract measures of effect or association, and prevalence data. Disease-specific and patient-relevant outcomes will be collected, including descriptions and severity of adverse events and the number of individuals affected by adverse events from the COVID-19 vaccines.

Additionally, we will extract information about the tools or instruments used to appraise the strength of the evidence as reported in primary studies described in the systematic reviews (e.g., Cochrane tool for risk of bias, Jadad scale, Newcastle-Ottawa scale or its adapted version, etc.), whether or not the authors conducted sensitivity analyses to determine the influence of low-quality primary studies, and the reported quality or risk of bias assessment.

We will import the cohort of systematic reviews to EndNote to facilitate the data management. To avoid misinterpretation of the extracted data, we will copy and paste the data directly from the systematic review into the investigators’ data extraction sheet. We would describe the number of studies that have data on each of the characteristics and the quality and strength of the evidence.

### Synthesis of the evidence

We will describe the collected data quantitatively with frequencies. Additionally, we will use the qualitative data description methods proposed by Arksey and O’Malley [29] to report the data narratively about the participants, journal characteristics, and the effectiveness and safety of COVID-19 vaccines in tables. For example, the column headings will contain the specific quantitative and qualitative variables we plan to collect including COVID-19 vaccine type, data on vaccine effectiveness, descriptions of vaccine-related adverse events, and the number of individuals affected by vaccine-related adverse events. (At the same time, the row headings will contain the specific descriptions or values for the corresponding columns. Specifically, the tables for the data extraction will contain, for example, adverse events as a column title, and the corresponding rows would list all adverse events found (rash, fever, etc.) from the systematic reviews, copied, and pasted directly into the data extraction sheet. The reviewers would then populate each cell for adverse event vs. rash with the number of systematic reviews that listed “rash” as an adverse event. When more than one systematic review describes the same information, we will extract the data from the most recent systematic review based on publication date followed by using the review authors’ adjudication of the quality of the studies that comprised their review rather than our judgement of quality. We will present the data synthesized in tables, to describe the data from the primary studies.

## Discussion

We plan to conduct the proposed scoping review using transparent reporting guided by evidence-based guidelines to ensure transparency and replicability of our search. In addition, we will collect qualitative evidence, which will provide valuable insights into the nature of the data in systematic reviews about COVID-19 vaccines and vaccine-associated adverse events. Any deviations from our proposed literature search and strategy, identification and selection of reviews, data collection and extraction, and evidence synthesis will be updated in the protocol and described in detail in the final draft of the scoping review.

Since the published literature on COVID-19 vaccines and related adverse events is constantly emerging, our proposed scoping review will provide an overview of the quality and availability of information to assess its accuracy. In this era of uncertainty with potentially misleading or harmful information surrounding COVID-19, the Internet can be a valuable tool to help the lay public access and use beneficial and relevant information to guide their healthcare decisions [30–48]. Misinformation about COVID-19 has led to harmful consequences [49]. Thus, the results of our review can be a source of the most up-to-date, accurate information on COVID-19 vaccination. Our results will supplement existing data about COVID-19 vaccines and their associated adverse events and newly add to the literature a summary of the evidence to provide researchers and the lay public a source of accurate information in the backdrop of continuously developing information about COVID-19 vaccines and their safety and effectiveness.

To provide the results of the proposed scoping review to as wide an audience as possible, especially to the lay public and researchers, we plan to publish the results of our review in a peer-reviewed, open-access journal. To disseminate further the results of our scoping review, we plan to present them at international conferences and meetings.

### Strengths of the proposed scoping review

This proposed scoping review is focused on summarizing the results of high-quality systematic reviews to assess the accuracy of the evidence concerning COVID-19 vaccines and their associated adverse events. Our scoping review would be the first to comprehensively assess the quality of available evidence in systematic reviews with or without meta-analyses about the effectiveness of and adverse-events associated with COVID-19 vaccines. Our assessment would highlight the accuracy of the evidence to inform decision-making concerning COVID-19 vaccines and their effectiveness and related adverse events.

## Supporting information

S1 File Checklist of the Preferred Reporting Items for Systematic Reviews and Meta-Analyses Protocols (PRISMA-P) statement adapted for a scoping review protocol. (DOCX)

S2 File Search strategy for Epistemonikos, Scopus, MEDLINE, CINAHL, CENTRAL, Web of Science Clarivate (Advanced search), WHO COVID-19 database, and the Joanna Briggs Institute of Excellence electronic databases. (DOCX)
