# Supplementary material for: Protocol of a scoping review of systematic reviews and meta-analyses about COVID-19 vaccines and associated adverse events from vaccination
Source: PLoS One. 2023 May 10;18(5):e0285442. doi: 10.1371/journal.pone.0285442 (PMC10171674; doi:10.1371/journal.pone.0285442)
Supplement: S2 File — (DOCX) [file pone.0285442.s002.docx]

S2 File. Search strategy for Epistemonikos, Scopus, MEDLINE, CINAHL, CENTRAL, Web of Science (Advanced search), WHO COVID-19 database, and the Joanna Briggs Institute of Excellence.

The search strategy for each database is listed below (search dates will be from January 1, 2019 to the present).

(1) Epistemonikos COVID-19 evidence using the custom year range set to 2019 to 2022 (September 16 was the date of the search) for all systematic reviews as the publication type in Cochrane reviews, Pubmed central, and systematic review question using the search term “Wuhan coronavirus” AND (vaccine OR vaccination OR immunise OR immunize OR immunising OR immunisation OR immunizing OR immunization))) in the title or abstract of the systematic review.

(2) Scopus – 0 (COVID-19) AND (randomised controlled trial OR observational studies)  AND (vaccine OR vaccination OR immunisation OR immunization OR revaccination OR vaccines OR vaccin* OR “adverse events”) AND NOT (animal or animals) AND (humans) AND ( LIMIT-TO ( PUBYEAR,2022) OR LIMIT-TO ( PUBYEAR,2021) OR LIMIT-TO ( PUBYEAR,2020) OR LIMIT-TO (PUBYEAR, 2019) AND ( LIMIT-TO ( DOCTYPE,"re" ) ) AND ( LIMIT-TO ( LANGUAGE,"English" ) ) AND ( LIMIT-TO ( SRCTYPE,"j" ) )

( covid-19 )  AND  ( clinical  AND trial )  AND  (vaccine OR vaccination OR immunise OR immunize OR immunising OR immunisation OR immunizing OR immunization OR “adverse events”)  AND NOT  ( animal  OR  animals )  AND  ( humans )  AND  ( LIMIT-TO ( SRCTYPE ,  "j" ) )  AND  ( LIMIT-TO ( DOCTYPE ,  "re" ) )  AND  ( LIMIT-TO ( PUBYEAR ,  2022 )  OR  LIMIT-TO ( PUBYEAR ,  2021 )  OR  LIMIT-TO ( PUBYEAR ,  2020 ) )  AND  ( LIMIT-TO ( LANGUAGE ,  "English" ) )  AND  ( LIMIT-TO ( EXACTKEYWORD ,  "Human" ) )

(3) MEDLINE -

1.        "Wuhan coronavirus".mp. or exp SARS-CoV-2/

2.        "COVID2019".mp. or exp COVID-19/

3.        "2019 ncov".ti,ab.

4.        (("novel coronavirus" or "new coronavirus") and (wuhan or "2019")).ti,ab.

5.        "2019nCoV".mp. or "2019nCoV".af.

6.        (wuhan and coronavirus).ti,ab.

7.        1 or 2 or 3 or 4 or 5 or 6

8.        (2020 Jan* or 2020 01* or 202001*).dp,ep.

9.        7 and 8

10.      randomized controlled trial.pt. or random*.ti. or (random* and (trial or placebo)).ti,ab.

11.      ['randomized controlled trial'/exp or 'single blind procedure'/exp or 'double blind procedure'/exp or 'crossover procedure'/]

12.      (random* or placebo* or factorial* or crossover* or 'cross-over' or 'cross over' or assign* or allocat* or volunteer* or ((singl* or doubl*) adj2 (blind* or mask*))).ab,ti.

13.      exp Epidemiologic Studies/ or exp Case Control Studies/ or exp Cohort Studies/ or exp Cross Sectional Studies/

14.      (case control or (cohort adj (study or studies)) or cross sectional or cohort analy$ or (follow up adj (study or studies)) or longitudinal or retrospective$ or prospective$).ab,ti. or (observ$ adj3 studies).mp. [mp=title, abstract, original title, name of substance word, subject heading word, floating sub-heading word, keyword heading word, organism supplementary concept word, protocol supplementary concept word, rare disease supplementary concept word, unique identifier, synonyms]

15.      (systematic review or meta-analysis or review).pt. or (review or meta-analysis or metaanalysis).ti.

16.      (comment or letter or editorial or news).mp. [mp=title, abstract, original title, name of substance word, subject heading word, floating sub-heading word, keyword heading word, organism supplementary concept word, protocol supplementary concept word, rare disease supplementary concept word, unique identifier, synonyms]

17.      (10 or 11 or 12 or 13 or 14 or 15) not 16

18.      immunization.mp.

19.      immunisation.mp.

20.      vaccines.mp.

21.      vaccination.mp.

22.      exp Vaccine/

23.      exp Vaccination/

24.      exp revaccination/

25.      vaccin*.ti,ab.

26.      revaccin*.ti,ab.

27.      immuniz*.ti,ab.

28.      immunis*.ti,ab.

29.      (vaccin* or inocul* or immuni*).tw.

30.      adverse events.mp.

31.      (adverse* adj3 (vaccin* or inocul* or immuni*)).mp.

32.      (adverse* adj1 (vaccin* or inocul* or immuni*)).ti,ab,kw.

33.      (safe or safety).tw. or side effect$.tw. or ((adverse or undesirable or harms$ or serious or toxic) adj3 (effect$ or reaction$ or event$ or outcome$)).tw. or exp product surveillance, postmarketing/ or exp adverse drug reaction reporting systems/

34.      18 or 19 or 20 or 21 or 22 or 23 or 24 or 25 or 26 or 27 or 28 or 29 or 30 or 31 or 32 or 33

35.      7 and 17 and 34

36.      limit 35 to (english language and yr="2019 -Current")

37.      "human".mp. [mp=title, abstract, original title, name of substance word, subject heading word, floating sub-heading word, keyword heading word, organism supplementary concept word, protocol supplementary concept word, rare disease supplementary concept word, unique identifier, synonyms]

38.      "human experiment".mp.

39.      37 or 38

40.      ("animal" or "animal experiment").mp. [mp=title, abstract, original title, name of substance word, subject heading word, floating sub-heading word, keyword heading word, organism supplementary concept word, protocol supplementary concept word, rare disease supplementary concept word, unique identifier, synonyms]

41.      (rat or rats or mouse or mice or murine or rodent or rodents or hamster or hamsters or pig or pigs or porcine or rabbit or rabbits or animal or animals or dogs or dog or cats or cow or bovine or sheep or ovine or monkey or monkeys).mp. [mp=title, abstract, original title, name of substance word, subject heading word, floating sub-heading word, keyword heading word, organism supplementary concept word, protocol supplementary concept word, rare disease supplementary concept word, unique identifier, synonyms]

42.      40 or 41

43.      (36 and 39) not "40".mp. [mp=title, abstract, original title, name of substance word, subject heading word, floating sub-heading word, keyword heading word, organism supplementary concept word, protocol supplementary concept word, rare disease supplementary concept word, unique identifier, synonyms]

(4) CINAHL –

#         Query

S54     S31 Not S53

S53     S32 OR S33 OR S34 OR S35 OR S36 OR S37 OR S38 OR S39 OR S40 OR S41 OR S42 OR S43 OR S44 OR S45 OR S46 OR S47 OR S48 OR S49 OR S50 OR S51 OR S52

S52     "monkey"

S51     "bovine" OR (MH "Cattle")

S50     "bovine or sheep" OR (MM "Sheep")

S49     "ovine"

S48     (MM "Sheep") OR "bovine or sheep or ovine"

S47     "cows" OR (MM "Cattle")

S46     (MH "Cats") OR "cats"

S45     (MM "Dogs") OR "dog or dogs"

S44     (MM "Rabbits") OR "rabbit or rabbits"

S43     (MM "Guinea Pigs") OR "porcine or pig"

S42     "pig or pigs" OR (MM "Guinea Pigs")

S41     (MM "Hamsters")

S40     (MH "Rodents") OR (MM "Rats") OR "rodent or rodents" OR (MM "Mice")

S39     "murine

S38     "mouse model or animal model or rat model"

S37     "mouse model or animal model or rat model"

S36     (MM "Mouse (Computer)") OR (MM "Mice") OR (MM "Bats")

S35     (MM "Rats") OR "rats"

S34     "animals, testing, experimentation" OR (MM "Animals+") OR (MM "Animals, Laboratory")

S33     MH animal experimentation

S32     MH animals

S31     S26 AND S29 AND S30

S30     S4 AND S5

S29     S27 OR S28

S28     TI ( human or humans ) OR AB ( human or humans )

S27     (MM "Human") OR "human"

S26     S6 OR S7 OR S8 OR S9 OR S10 OR S11 OR S12 OR S13 OR S14 OR S15 OR S16 OR S17 OR S18 OR S19 OR S20 OR S21 OR S22 OR S23 OR S24 OR S25

S25     AB vaccin*

S24     TI vaccin*

S23     AB immuni*

S22     TI immuni*

S21     AB (vaccin* N3 (safe or reaction or side effect* or adverse or undesirable or harms* or serious or toxic or reaction*))

S20     TI (vaccin* N3 (safe or reaction or side effect* or adverse or undesirable or harms* or serious or toxic or reaction*))

S19     TI ((vaccin* or immuni*) N1 (adverse events)) OR AB ((vaccin* or immuni*) N1 (adverse events))

S18     TI ((vaccin* or immuni*) N1 sequelae)) OR AB ((vaccin* or immuni*) (sequelae))

S17     (MM "Respiratory System Abnormalities" OR "physical abnormalities") N1 (adverse events))

S16     TX (Serious events) OR TX (Toxic events)

S15     "adverse events in healthcare" OR TX (side effects) OR TX (harmful effects)

S14     (MM "Adverse Health Care Event")

S13     TX (adverse effects )

S12     MH ("harmful effects")

S11   MH vaccination

S9     (MH Immunisation+)

S8     (MH Immunization+)

S7      (MH Vaccine)

S6      (MH Vaccines)

S5      (MM "Randomized Controlled Trials+") OR (MM "Clinical Trials+") OR "randomized controlled trials or rtc or randomised control trials or randomized clinical trial or randomized controlled study" OR (MH "Case Control Studies+") OR (MH "Case Studies") OR (MH "Cross Sectional Studies") OR (MH "Prospective Studies+") OR (MH "Retrospective Panel Studies") OR (MH "Correlational Studies") OR (MH "Ecological Research") OR (MH "Descriptive Research") OR TI epidemiologic OR AB epidemiologic OR TI "case control" OR TI “case referent” OR AB “case referent*” OR TI “case stud*” OR AB “case stud*” OR TI “case series” OR AB “case series” OR TI cohort* OR AB cohort* OR TI “cross sectional” OR AB “cross sectional” OR TI “follow up” OR AB “follow up” OR TI longitudinal OR AB longitudinal OR TI retrospective* OR AB retrospective* OR TI prospective* OR AB prospective* OR TI observational OR AB observational OR TI “Controlled before and after” OR AB “Controlled before and after” OR TI “Interrupted time series” OR AB “Interrupted time series”

S4       S1 OR S2

S3       S1 OR S2

S2       TI ( wuhan and coronavirus ) OR AB ( wuhan and coronavirus )

S1       (MM "SARS-CoV-2") OR (MH "Coronavirus Infections+") OR (MH "Coronavirus+") OR (MM "COVID-19") OR ""Wuhan coronavirus""

(5) CENTRAL

#1       ("Wuhan coronavirus".mp. or exp SARS-CoV-2/):kw (Word variations have been searched)

#2       "COVID2019"

#3       COVID-19

#4       ("2019 ncov"):ti,ab,kw

#5       (("novel coronavirus" or "new coronavirus") and (wuhan or "2019")):ti,ab,kw

#6       (wuhan and coronavirus):ti,ab,kw

#7       "Wuhan coronavirus"

#8       #21 or #22 or #23 or #24 or #25 or #26

#9       mh Vaccines OR mh Vaccination OR "biological abnormalities" OR (vaccin* or revaccin* or inocul* or immuni*):ti,ab OR “adverse effects” OR symptoms OR pathophysiology OR sequelae

#10     randomised trial or (clinical NEAR/2 trial*):ti,ab

#11     epidemiologic:ab,ti

#12     (“case control” OR “case stud*” OR “case series” OR Correlational OR “ecological stud*” OR “Descriptive stud*”):ab,ti

#13     (cohort* OR “follow up” OR longitudinal OR retrospective* OR prospective* OR observational OR “Controlled before and after” OR “Interrupted time series”):ab,ti

#14     #29 OR #30 OR #31 OR #32

#15     #27 and #28 and #29

#16     (animal or animals or rat or rats or mouse or mice or murine or rodent or rodents or hamster or hamsters or pig or pigs or porcine or rabbit or rabbits or animal or animals or dogs or dog or cats or cow or bovine or sheep or ovine or monkey or monkeys) OR AB=(animal or animals or rat or rats or mouse or mice or murine or rodent or rodents or hamster or hamsters or pig or pigs or porcine or rabbit or rabbits or animal or animals or dogs or dog or cats or cow or bovine or sheep or ovine or monkey or monkeys)

#17     #34 not #35

(6) Web of Science Clarivate (Advanced search)

#1 TI=(COVID OR COVID19 OR "SARS‐CoV‐2" OR "SARS‐CoV2" OR SARSCoV2 OR"SARSCoV‐2" OR "SARS coronavirus 2" OR "2019 nCoV" OR "2019nCoV" OR "2019‐novel CoV" OR "nCov 2019" OR "nCov 19" OR "severe acute respiratory syndrome coronavirus 2" OR "novel coronavirus disease" OR "novel corona virus disease" OR "corona virus disease 2019" OR "coronavirus disease 2019" OR "novel coronavirus pneumonia" OR "novel corona virus pneumonia" OR "severe acute respiratory syndrome coronavirus 2") OR AB=(COVID OR COVID19 OR "SARS‐CoV‐2" OR "SARS‐CoV2" OR SARSCoV2 OR"SARSCoV‐2" OR "SARS coronavirus 2" OR "2019 nCoV" OR "2019nCoV" OR "2019‐novel CoV" OR "nCov 2019" OR "nCov 19" OR "severe acute respiratory syndrome coronavirus 2" OR "novel coronavirus disease" OR "novel corona virus disease" OR "corona virus disease 2019" OR "coronavirus disease 2019" OR "novel coronavirus pneumonia" OR "novel corona virus pneumonia" OR "severe acute respiratory")

#2 TI=(vaccination OR Immunisation OR Immunization OR vaccines OR vaccine OR vaccination OR immunise OR immunize OR immunising OR immunizing OR "adverse events" OR symptoms OR "biological abnormalities" OR pathophysiology OR sequelae) OR AB=(vaccination OR Immunisation OR Immunization OR vaccines OR vaccine OR vaccination OR immunise OR immunize OR immunising OR immunizing OR "adverse events" OR “side effects” OR "biological abnormalities" OR “toxic effects” OR “serious effects”)

#3 TI=(randomised trial OR cohort* OR “follow up” OR longitudinal OR retrospective* OR prospective* OR observational OR “Controlled before and after” OR “Interrupted time series OR case control” OR “case stud*” OR “case series” ) OR AB=(randomised trial OR cohort* OR “follow up” OR longitudinal OR retrospective* OR prospective* OR observational OR “Controlled before and after” OR “Interrupted time series OR case control” OR “case stud*” OR “case series” )

#4 TI=(human or humans) OR AB=(human or humans)

#5 TI=(animal or animals or rat or rats or mouse or mice or murine or rodent or rodents or hamster or hamsters or pig or pigs or porcine or rabbit or rabbits or animal or animals or dogs or dog or cats or cow or bovine or sheep or ovine or monkey or monkeys) OR AB=(animal or animals or rat or rats or mouse or mice or murine or rodent or rodents or hamster or hamsters or pig or pigs or porcine or rabbit or rabbits or animal or animals or dogs or dog or cats or cow or bovine or sheep or ovine or monkey or monkeys)

#6 #1 AND #2 AND #3 AND #4

#6 NOT #5

#6 NOT #5 and Review Articles or Articles (Document Types) and Publication Years: 2019 or 2020 or 2021

(7) WHO COVID-19 database

 (tw:((tw:((tw:(COVID-19)) OR (tw:(Wuhan coronavirus)) OR (tw:(SARS-CoV-2)) AND (tw:(vaccine)) OR (tw:(vaccination)) OR (tw:(immunise)) OR (tw:(immunize)) OR (tw:( immunization)) OR (tw:(immunisation)) OR (tw:(immunizing)) OR (tw:(immunising)) OR (tw:(adverse events)) OR (tw:((physical or biological) abnormalities)) OR (tw:(symptoms)))) AND (tw:(human)) AND NOT (tw:(animals )))) AND (tw:((randomised or randomized) trial))  OR Epidemiologic Studies OR Case‐Control Studies OR Cohort Studies OR Cross‐Sectional Studies OR longitudinal OR retrospective* OR prospective*

type:("article") AND mj:("COVID-19" OR "Coronavirus Infections" OR "SARS-CoV-2" OR "COVID-19 Vaccines" OR "Coronavirus" OR "Respiratory Distress Syndrome" OR "Social Media" OR "Severe Acute Respiratory Syndrome") AND type_of_study:("guideline" OR "systematic_reviews" OR "overview" OR "sysrev_observational_studies") AND la:("en") AND clinical_aspect:("prognosis" OR "diagnosis" OR "therapy") AND year_cluster:("2021" OR "2020" OR "2019")

(8) Campbell Collaboration using the custom year range set to 01-01-2019 to 07-04-2023 for all systematic reviews as the publication type using the search term “COVID”.

(9) Joanna Briggs Institute of Excellence using the custom year range set to 2019 to 2023 for all systematic reviews using the search term “COVID”.
