# Supplementary material for: Protocol of a scoping review of systematic reviews and meta-analyses about COVID-19 vaccines and associated adverse events from vaccination
Source: PLoS One. 2023 May 10;18(5):e0285442. doi: 10.1371/journal.pone.0285442 (PMC10171674; doi:10.1371/journal.pone.0285442)
Supplement: Attachment — Submitted filename: Response to reviewers.docx [file pone.0285442.s003.docx]

Reviewers' comments: Reviewer's Responses to Questions

**Comments to the Author**

1. Does the manuscript provide a valid rationale for the proposed study, with clearly identified and justified research questions? The research question outlined is expected to address a valid academic problem or topic and contribute to the base of knowledge in the field.

Reviewer #1: Partly

Reviewer #2: Yes

Response 1: We thank the Reviewers for bringing our attention to the fact that our research question warrants clarification. We have now revised our research question now on lines 125-126 to “what is the available evidence on COVID-19 vaccines and associated adverse events from COVID-19 vaccines reported in systematic reviews and meta-analyses?” We have additionally provided a revised rationale for the revised research question on lines 131-136 that states, “As the development of vaccines has come to the forefront in preventing COVID-19, we have chosen to focus our scoping review on vaccines and adverse events arising from their use. This research question would guide our scoping review to summarize the evidence on the growing body of systematic reviews on COVID-19 vaccines and related adverse events. Further, a comprehensive summary of systematic reviews of the evidence about COVID-19 vaccines and their reported adverse events would provide descriptions of their effectiveness and safety to inform the public and policy makers.”

2. Is the protocol technically sound and planned in a manner that will lead to a meaningful outcome and allow testing the stated hypotheses? The manuscript should describe the methods in sufficient detail to prevent undisclosed flexibility in the experimental procedure or analysis pipeline, including sufficient outcome-neutral conditions(e.g. necessary controls, absence of floor or ceiling effects) to test the proposed hypotheses and a statistical power analysis where applicable. As there may be aspects of the methodology and analysis which can only be refined once the work is undertaken, authors should outline potential assumptions and explicitly describe what aspects of the proposed analyses, if any, are exploratory.

Reviewer #1: Partly

Reviewer #2: No

Response 2: Thank you for providing comments on the refinement needed for the overall robustness and clarity of the methodology. We have now followed both Reviewers’ specific comments, which we addressed below to clarify the objectives, research question, current state of the protocol, and specifics surrounding data extraction and collection accordingly to provide more sufficient details for undertaking the scoping review.

3. Is the methodology feasible and described in sufficient detail to allow the work to be replicable? Descriptions of methods and materials in the protocol should be reported in sufficient detail for another researcher to reproduce all experiments and analyses. The protocol should describe the appropriate controls, sample size calculations, and replication needed to ensure that the data are robust and reproducible.

Reviewer #1: Yes

Reviewer #2: No

Response 3: We are grateful for the suggested changes to the methods suggested in the detailed Reviewers’ comments. Based on both Reviewers’ specific comments regarding the needed changes to the objectives, research question, and methodology, we have now provided focused and clearer objectives, a refined research question based on the revised objectives, and expanded parts of the methodology that were vague.

4. Have the authors described where all data underlying the findings will be made available when the study is complete? The PLOS Data policy requires authors to make all data underlying the findings described in their manuscript fully available without restriction, with rare exception, at the time of publication. The data should be provided as part of the manuscript or its supporting information, or deposited to a public repository. For example, in addition to summary statistics, the data points behind means, medians and variance measures should be available. If there are restrictions on publicly sharing data—e.g. participant privacy or use of data from a third party—those must be specified.

Reviewer #1: No

Reviewer #2: No

Response 4: We thank the Reviewers for bringing our attention to the fact that it is unclear whether we will make the findings publicly available when the study is complete. We already have the protocol posted to the Open Science Framework (OSF) and we have now made our existing Data Availability Statement on p. 14, lines 342-344, more explicit by stating that “The study data, protocol, extraction sheets, and search strategy will be made available on the Open Science Framework when the study is complete at<https://osf.io/gkv74/?view_only=f93fe736a60747e09d3c7d442a49e1bc>. Additionally, we have now added a sentence from lines 145-146 that explicitly state that “Our methods were described in detail in our protocol posted on Open Science Framework (https://osf.io/gkv74/?view_only=f93fe736a60747e09d3c7d442a49e1bc).”

5. Is the manuscript presented in an intelligible fashion and written in standard English? PLOS ONE does not copyedit accepted manuscripts, so the language in submitted articles must be clear, correct, and unambiguous. Any typographical or grammatical errors should be corrected at revision, so please note any specific errors here.

Reviewer #1: Yes

Reviewer #2: No

Response 5: We realize that the protocol could be written in a clearer, more explicit manner. We have now edited the manuscript for clarity according to the suggestions provided by the Reviewers, thank you.

6. Review Comments to the Author

Please use the space provided to explain your answers to the questions above and, if applicable, provide comments about issues authors must address before this protocol can be accepted for publication. You may also include additional comments for the author, including concerns about research or publication ethics. You may also provide optional suggestions and comments to authors that they might find helpful in planning their study. (Please upload your review as an attachment if it exceeds 20,000 characters)

Reviewer #1: Most concerning is the ambiguous question that deals with many different aspects ofCovid-19. It isn't clear how all these will be dealt with in a single umbrella review or how the authors hope to tie them together. In a review, everything hangs or falls on the question, so until this is stated more clearly and concisely the rest is difficult to determine whether it will be successful. See line references below for additional suggested comments.

Response: We thank the Reviewers for bringing our attention to the fact that our research question warrants clarification. We have now shortened the scoping review question, omitting disparate aspects of COVID-19. We have now revised our research question now on lines 125-126 to “what is the available evidence on COVID-19 vaccines and associated adverse events from COVID-19 vaccines reported in systematic reviews and meta-analyses?” We have additionally provided a revised rationale for the revised research question on lines 131-136 that states, “As the development of vaccines has come to the forefront in preventing COVID-19, we have chosen to focus our scoping review on vaccines and adverse events arising from their use. This research question would guide our scoping review to summarize the evidence on the growing body of systematic reviews on COVID-19 vaccines and related adverse events. Further, a comprehensive summary of systematic reviews of the evidence about COVID-19 vaccines and their reported adverse events would provide descriptions of their effectiveness and safety to inform the public and policy makers.”

Line References 31 It is not clear if you will be looking at just other reviews, or also RCTs and observational trials as well. This is clearer in your full text (lines 152)

Response: We have now clarified which sources we will be included and excluded on lines 43-44 and 199-200. We have now explicitly stated that we will exclude individual randomized controlled trials or observational studies.

32-35 a very long list of outcomes for this review. will you be able to adequately look at all of these?

Response: Thank you for bringing our attention to the long list of outcomes for the scoping review. We agree with the Reviewer about reducing the outcomes, thus we have now reduced the number of outcomes to only include COVID-19 vaccines and associated vaccine adverse events, which we now stated throughout the manuscript.

35-37 include platforms as well as databases

Response: We have now stated that the platform will be Ovid for searching in the MEDLINE, CINAHL, and CENTRAL databases in the abstract on lines 40-41, in the main text on lines 156, and on lines 169-170.

95 PRISMA does not provide guidance but is simply a list of items that should be reported on

Response: We thank the Reviewer for the helpful suggestion. We have now revised our description of our use of the PRISMA-P from lines 114 to 115 we have now stated that “We reported this scoping review in accordance to the Preferred Reporting Items for Systematic Reviews…”, rather than describing that the PRISMA-P offered guidance in the reporting of the protocol.

127-129 provide platform names as well as database names (eg. Ovid Medline or Ebsco Medline)

Response: We are thankful to the Reviewer for noticing that the platform names are not provided. Similar to Response 4, we have now provided platform names in the previously mentioned parts of the manuscript (on lines 40-41, in the main text on lines 156, and on lines 169-170).

204 tracking the impact factor of the journal is a new idea for me. Curious why this would be needed? Would this privilege some disciplines over others? for example, medical journals almost always have a higher impact factor than nursing journals. or oncology journals have a higher impact factor than infectious diseases.

Response: The impact factor of journals would be collected solely as a characteristic of the journals in which the studies are published to inform readers about this particular metric. We did not intend to further analyze the impact factor or categorize the studies according to the impact factor. To clarify our intention to collect the impact factor for purely descriptive purposes, line 241 now includes the text, “… journal-level characteristics including journal title and impact factor…”

240-241 higher quality according to whom? the authors of this paper or the authors of the initial review. not clear.-

Response: We thank the Reviewer for highlighting the unclear description of how we will select between more than one systematic review that describes the same information. To be clearer, we now state on lines 287-288 that we would use the review authors’ adjudication of the quality of the studies that comprised their review rather than our judgement of quality.

Reviewer #2: I commend the vision of the authors to conduct an updated and comprehensive systematic review.

1. They should think carefully and provide justifications for the methods from selecting, retrieving recent publications up until the process of assessing risk bias and reporting the results for each of the following objective: i) disease management ii) management of COVID-19 iii) risk factor iv) diagnostic methods v) disease outcomes vi) preventive measures vii) adverse events from treatments viii)adverse events from vaccines ix) modes of disease transmission

Response: We thank the Reviewer for suggesting that we provide justifications for selecting publications with the stated objectives. However, following advice from Reviewer #1, we have now revised the previous objectives. We have now revised our research question now on lines 125-126 to “what is the available evidence on COVID-19 vaccines and associated adverse events from COVID-19 vaccines reported in systematic reviews and meta-analyses?” We have additionally provided a revised rationale for the revised research question on lines 131-136 that states, “As the development of vaccines has come to the forefront in the prevention of COVID-19, we have chosen to shift the focus of our scoping review to vaccines and adverse events arising from their use. This research question would guide our scoping review to summarize the evidence on the growing body of systematic reviews on COVID-19 vaccines and related adverse events. Further, a comprehensive summary of systematic reviews of the evidence about COVID-19 vaccines and their reported adverse events would provide descriptions about their effectiveness to inform the public and policy makers.”

2. Research questions need to be specified and detailed more than:

“what is the available evidence is on symptoms, treatments, risk factors, diagnostic methods, the target population for treatments, the target population for vaccines, goals of the treatment, goals of vaccination, disease outcomes, vaccination, preventive measures, adverse events from treatment, adverse events from vaccines, the pathophysiology, and modes of transmission for COVID-19 to establish the quality and accuracy of the information?”

Response: We are grateful for the comments about the research question. Since we have now focused on COVID-19 vaccines and COVID-19 vaccine-related adverse events, our research question has thus become clearer and more detailed as “what is the available evidence on COVID-19 vaccines and associated adverse events from COVID-19 vaccines reported in systematic reviews and meta-analyses?

3. Including publications from January 2020 from the point of outbreak defeats the purpose of finding recent evidence.

Response: We understand the Reviewers’ concerns about the limited search dates for this study. However, we chose this date based on when the WHO declared COVID-19 a pandemic in January 2020, thus we intended to make the search as focused as possible. To allow for a more comprehensive search, we have now included 2019 as the initial year of the search to extend the dates to before the pandemic. We have now revised the initial search year to January 2019 on lines 39, 152 and 497. Accordingly, the search strategies have now been revised to reflect this change in S2 File.

4. The extraction and process for creating results tables will need to be specified with details on the column headings.

Response: We have now provided specific details about the column headings on lines 271-285. We have specified the description to “For example, the column headings will contain the specific quantitative and qualitative variables we plan to collect including COVID-19 vaccine type, data on vaccine effectiveness, descriptions of vaccine-related adverse events, and the number of individuals affected by vaccine-related adverse events.”

Lines 202-205 are too general and does not elude to the objectives listed in item1.

Response: We thank the Reviewer for noticing the lack of detail in our description about the data collection and extraction surrounding our objectives. Accordingly, we have now revised the text in the “Data collection and extraction” section to elude to the objectives of the study, now described on lines 237-240 as “Aligned with the objectives concerning gathering evidence for this scoping review, all reviewers will extract relevant data on COVID-19 vaccines and vaccine-related adverse events that will allow an assessment of the data to provide a subsequent comprehensive summary from the systematic reviews. We will extract data from systematic reviews, including the author, dates of publication (year), publication title…”

5. I would highly recommend to have the English language construct reviewed. As it stands, the manuscript does not make clear the current stage of the protocol.

Response: We have now revised the protocol to clarify the writing. Additionally, lines 177-178 now state the current stage of the protocol in the Methods section in the “Sources of information” section. Specifically, we now describe after the description of the databases to be searched that we have begun initial searches and have not proceeded to the study selection phase: “Regarding the current stage of this scoping review, we have only performed preliminary searches in the databases and have not proceeded to the study selection phase.”

7. PLOS authors have the option to publish the peer review history of their article (what does this mean?). If published, this will include your full peer review and any attached files. If you choose “no”, your identity will remain anonymous but your review may still be made public. **Do you want your identity to be public for this peer review?** For information about this choice, including consent withdrawal, please see our Privacy Policy.

Reviewer #1: **Yes:** Amanda Ross-White

Reviewer #2: N
